# Evaluating the Effects of Temperature on Mortality in Manila City (Philippines) from 2006–2010 Using a Distributed Lag Nonlinear Model

**DOI:** 10.3390/ijerph120606842

**Published:** 2015-06-16

**Authors:** Xerxes T. Seposo, Tran Ngoc Dang, Yasushi Honda

**Affiliations:** 1 Graduate School of Comprehensive Human Sciences, University of Tsukuba, Tsukuba City, Ibaraki Prefecture 305-8577, Japan; E-Mail: ngocdangytcc@gmail.com; 2 Department of Environmental Health, Faculty of Public Health, University of Medicine and Pharmacy, Ho Chi Minh City 70000, Vietnam; 3 Faculty of Health and Sports Sciences, University of Tsukuba, Tsukuba City, Ibaraki Prefecture 305-8577, Japan; E-Mail: honda@taiiku.tsukuba.ac.jp

**Keywords:** temperature-mortality relationship, distributed lag nonlinear model, category-specific mortality, all-cause mortality

## Abstract

The effect of temperature on the risk of mortality has been described in numerous studies of category-specific (e.g., cause-, sex-, age-, and season-specific) mortality in temperate and subtropical countries, with consistent findings of U-, V-, and J-shaped exposure-response functions. In this study, we analyzed the relationship between temperature and mortality in Manila City (Philippines), during 2006–2010 to identify the potential susceptible populations. We collected daily all-cause and cause-specific death counts from the Philippine Statistics Authority-National Statistics Office and the meteorological variables were collected from the Philippine Atmospheric Geophysical and Astronomical Services Administration. Temperature-mortality relationships were modeled using Poisson regression combined with distributed lag nonlinear models, and were used to perform cause-, sex-, age-, and season-specific analyses. The minimum mortality temperature was 30 °C, and increased risks of mortality were observed per 1 °C increase among elderly persons (RR: 1.53, 95% CI: 1.31–1.80), women (RR: 1.47, 95% CI: 1.27–1.69), and for respiratory causes of death (RR: 1.52, 95% CI: 1.23–1.88). Seasonal effect modification was found to greatly affect the risks in the lower temperature range. Thus, the temperature-mortality relationship in Manila City exhibited an increased risk of mortality among elderly persons, women, and for respiratory-causes, with inherent effect modification in the season-specific analysis. The findings of this study may facilitate the development of public health policies to reduce the effects of air temperature on mortality, especially for these high-risk groups.

## 1. Introduction

The effects of climate change on human health have received significant attention in recent years, which has led to an increased focus on how meteorological factors affect the mortality risk [1]. The association between temperature and mortality was first reported during the early 20th century, and ongoing research has attempted to continuously refine our understanding of this association by establishing the robustness of various models [2]. In addition, studies regarding the temperature-mortality relationship are becoming increasingly common throughout the world and in Southeast Asia, with various studies being conducted in the cities of Vietnam, Thailand, and Taiwan [3,4]. Furthermore, several researchers have reported that both high and low temperatures are associated with an increased risk of mortality. For example, these associations between the temperature extremes and risk of mortality have commonly been described through U-, V-, and J-shaped exposure-response functions [5,6]. In these functions, the temperature with the lowest risk of mortality is defined as the minimum mortality temperature (MMT), which varies for different geographical locations [7].

The relative risks (RR) for cause- and age-specific mortality at both temperature extremes are noticeably higher than at the MMT and nearby temperature percentiles [6,7]. Cause-specific mortality especially due to cardiovascular and respiratory diseases has different RRs for both temperature extremes [1,2,6,7,8,9,10]. In addition, temperature also affects people who are at-risk of adverse events, due to pre-existing cardiovascular-related and respiratory-related conditions [1,6]. Age-specific studies have also reported that the elderly population has a relatively high susceptibility to extreme temperatures [2,9]. In this context, the body is capable of a physiological response to temperature changes (thermoregulation), which enables a person to adapt to the changing temperature. However, the body’s thermoregulatory capacity decreases with age, which makes the elderly population more susceptible to extreme temperatures [1,8,10].

The risk due to the lag effect also varies according to the cause of death. For example, cardiovascular-related deaths are mostly observed for shorter lags, and respiratory-related deaths are prominent for longer lags [11]. The variability of these risks are also affected by several covariates, such as the day of the week, relative humidity, particulate matter (PM), seasonal variations, and holidays [2,6,7,8,10]. However, there are no definitive and conclusive data with regard to which sex experiences the greatest risk, as different studies have reported conflicting findings. Similarly, season-specific effects are only valid for the study area, as seasonal effets often varies across countries.

The government of the Philippines is in the process of crafting evidence-based policies that are aimed to inform the population and to identify clear early warning signs of an increased health-related risk. These risks and warning signs have been consistently supported by analyses of datasets from various countries across the globe. Therefore, in this study, we explored the plausibility of applying previously reported prominent risk signs (which have been identified in temperate and other countries) to risk prediction in the Philippines. This is the first study to analyze the temperature-mortality relationship in Manila City (Philippines) using daily data.

## 2. Materials and Methods

### 2.1. Study Site

Manila City (latitude 14°5′ N and longitude 120°8′ E) is the capital of the Philippines and is highly urbanized, with a population of 1.6 million in 2010 (51% of the population is male) [12]. In addition, the continuous expansion of the city has increased its industrialization via an influx of people who have relocated from the provinces. Manila City has a land area of 24.98 km^2^ and the highest population density (66,140 people per square kilometer) among the 17 cities and municipalities that comprise the National Capital Region.

### 2.2. Meteorological and Mortality Data

We collected data regarding meteorological variables, such as daily average temperature and daily average humidity, during 2006–2010 from the Philippine Atmospheric Geophysical and Astronomical Services Administration (PAGASA). We also collected data regarding the daily death counts during the same period from the Philippine Statistics Authority-National Statistics Office (PSA-NSO); these data were coded using the International Classification of Diseases (ICD) 10 system. Next, we categorized the mortality data according to cause, sex, age, and season, in order to analyze the specific susceptibilities to temperature in these categories. Cardiovascular-related mortality was defined using ICD codes I00–I99, and respiratory-related mortality was defined using ICD codes J00–J99. Age-specific mortality was grouped into 0–14 years old, 15–64 years old, and ≥65 years old. The season-specific mortalities were categorized using the PAGASA seasonal classifications: December-January-February (DJF) is the northeast monsoon season, March-April-May (MAM) is the summer season, June-July-August (JJA) is the southwest monsoon season, and September–October–November (SON) is the transitional period from the southwest to the northeast monsoon season [13].

### 2.3. Modeling Approach

We used Poisson generalized linear models with overdispersion for the time series data to evaluate the nonlinear relationship between temperature and mortality [14,15]. In the initial analysis, the temperature-mortality relationship was analyzed using a distributed lag nonlinear model (DLNM) with a natural cubic spline (NCS), as its smoothing parameter applied to both average temperature and lag dimensions; this model is referred to as the NCS-NCS model [8,9,15,16]. These splines relaxed the assumption of linearity for non-linear relationships, which enables better fitting of the model. The DLNM also simultaneously analyzes the relationship between the nonlinear pattern and the lagged effects, which makes it an ideal tool for modeling the relationship between temperature and mortality [17]. This flexibility in the analysis facilitates the simultaneous quantification of the lag effects and the temperature-mortality relationship modeling. In this study, we used different predictor space functions while maintaining the lag space with NCS, although there are other predictor space functions that can be used; these functions have been discussed previously [18].

**Figure 1:**
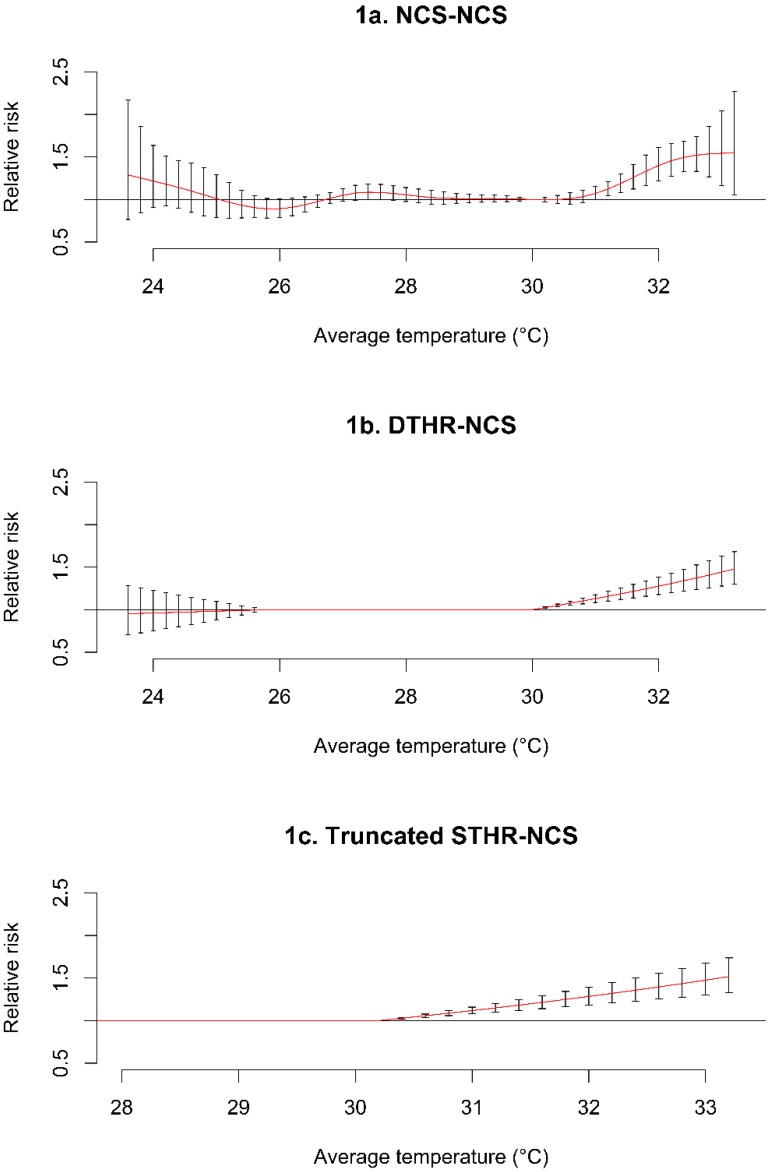
The model parameterization with NCS-NCS (**a**), DTHR-NCS (**b**), and truncated STHR-NCS (**c**); The DTHR-NCS thresholds were set at the two minimum points that were observed in NCS-NCS, while the upper threshold in the STHR-NCS was based on the best combination of the upper and lower threshold that minimized the lower temperature effect; NCS: natural cubic spline, STHR: single high threshold, DTHR: double high threshold.

After a series of steps to achieve model specification and simplification (Supplementary Materials), the final model of temperature and all-cause mortality was comprised of a hockey-stick model with a single high threshold (STHR) at 30.2 °C (see Figure 1c below). In this model, *β_High’_* serves as the vector of the regression coefficients for the new high threshold for the STHR-NCS model in Equation (1):

(1) $$Log\left[E\left(Y_{t}\right)\right]=\text{α}+\beta_{High^{\prime }}THig{h^{\prime }}_{t,l}+ns\left(date,7\times 5\right)+ns\left(RHave_{t},3\right)+as.factor\left(dow\right)+hod$$

For the cause-specific, sex-specific, age-specific, and season-specific analyses, we initially set each model to the NCS-NCS specification, and then objectively modified and simplified the models based on their respective MMTs, knots, and possible threshold points. Although not all the models had a double high threshold (DTHR)-NCS or STHR-NCS specification, all models were created using the same starting parameters in their NCS-NCS models. However, the season-specific analysis was treated differently in this study, as Gasparrini [18] has reported that ordered series that are equally-spaced for a specific season in respective years do not constitute a single continuous series, compared to the other analyses. Therefore, the season-specific analysis used different parameterization, which is described in Equation (2):

(2) $$Log\left[E\left(Y\prime_{season}\right)\right]=\alpha +\beta_{season}T_{season,l}+ns\left(doy_{season},4\right)+ns\left(time_{season},3\right)+ns\left(RHave_{season},3\right)+as.factor\left(dow_{season}\right)+hod_{season}$$

In this model, we followed the specifications of Gasparrini [19], and used the day of the year (*doyseason*) to control for the seasonal effect per year and time (*timeseason*) and to account for the long-term trend; all other terms are season-specific parameters. In the initial development, we observed extremely wide confidence intervals that suppressed the model pattern identification, and so we chose to use the log-transformed season-specific mortality ($Y\prime_{season}$).

## 3. Results

Table 1 shows the summary statistics for the meteorological and mortality variables in Manila City during the study period. There were 94,656 all-cause deaths during 2006–2010, with cardiovascular causes comprising 28.3% of these cases and respiratory causes comprising 12.4% of these cases. Mortality was more frequent among men (57.1%), compared to among women (42.9%). More than half (51.1%) of all mortalities occurred in the 15–64-year-old age group, compared to 32.1% in the ≥65-year-old age group and 16.8% in the 0–14-year-old age group. Manila City experienced a narrow temperature range throughout the year (23.5–33.3 °C), with the highest temperature being recorded during MAM and the lowest temperature being recorded during DJF and SON.

**Table 1:** A summary of the meteorological and mortality statistics in Manila city during 2006–2010.

Statistic	Mean	SD	Min	10th Percentile	50th Percentile	90th Percentile	Max
*Average temperature*	28.8	1.52	23.5	26.8	28.8	30.7	33.3
*Average relative humidity*	73.9	7.46	53.0	64.0	74.0	83.0	100
*Season-specific temperature*							
DJF	27.6	1.19	23.5	26.1	27.6	29.0	30.5
MAM	29.8	1.36	24.8	28.2	29.8	31.5	33.3
JJA	29.1	1.37	24.8	27.3	29.2	30.8	32.5
SON	28.6	1.15	23.5	27.0	28.7	29.9	31.5
All-cause mortality	52.0	8.00	14.0	42.0	52.0	63.0	81.0
*Cause-specific mortality*							
Cardiovascular	14.7	4.03	1.00	10.0	14.0	20.0	29.0
Respiratory	6.44	2.79	0.00	3.00	6.00	10.0	18.0
*Sex-specific mortality*							
Women	22.3	5.24	3.00	16.0	22.0	29.0	38.0
Men	29.7	5.98	7.00	22.0	29.0	37.0	53.0
*Age-specific mortality*							
0–14 years old	8.70	3.23	0.00	5.00	9.00	13.0	21.0
15–64 years old	26.5	5.68	6.00	20.0	26.0	34.0	48.0
≥65 years old	16.7	4.31	2.00	11.0	16.0	22.0	31.0
*Season-specific mortality*							
DJF	51.4	7.33	31.0	42.0	51.0	61.0	70.0
MAM	50.1	8.37	27.0	40.0	49.0	61.0	81.0
JJA	52.8	9.02	14.0	42.0	52.0	64.0	78.0
SON	53.3	8.53	15.0	43.0	53.0	64.0	75.0

Min: minimum, SD: standard deviation, Max: maximum, DJF: December to February, MAM: March to May, JJA: June to August, SON: September to November.

Table 2 shows the NCS-NCS RRs for category-specific mortality in the 1st, 5th, 95th and 99th temperature percentiles with their respective MMTs. The season-specific data were omitted from this analysis, due to the extremely wide confidence intervals, which might have been caused by effect modification. Higher temperature effects are prominent among the different risk groups in the 99th temperature percentile, with the ≥65 year old age group exhibiting the highest risk. Most of the MMTs occurred at 30.0 °C, which was at the 80th temperature percentile.

Figure 1a–c show the model transitions for the NCS-NCS, DTHR-NCS and STHR-NCS parameterizations of temperature and all-cause mortality. Figure 1a shows the NCS-NCS model with increasing risk in both tails and an MMT at 30.0 °C. Figure 1b assumes a DTHR-NCS model with threshold points set at the minimum points that were observed in Figure 1a, and Figure 1c shows a linear increase in the temperature and all-cause mortality relationship, with a new high threshold at 30.2 °C.

**Table 2:** The NCS-NCS RR of category-specific mortality in the 1st, 5th, 95th, and 99th temperature percentiles and respective MMTs.

Statistic	1st Percentile (RR_Fit_)	95% CI	5th Percentile (RR_Fit_)	95% CI	95th Percentile (RR_Fit_)	95% CI	99th Percentile (RR_Fit_)	95% CI	MMT (°C)
*All-cause mortality*	1.01	(0.79–1.29)	0.89	(0.79–1.01)	1.07	(1.00–1.15)	1.40	(1.22–1.61)	30
*Cause-specific mortality*									
Cardiovascular	1.32	(0.87–2.01)	1.17	(0.94–1.45)	1.15	(1.01–1.30)	1.37	(1.07–1.75)	30
Respiratory	0.88	(0.65–1.19)	0.77	(0.60–0.98)	1.16	(0.97–1.39)	1.52	(1.23–1.88)	29
*Sex-specific mortality*									
Women	1.05	(0.82–1.35)	0.96	(0.82–1.12)	1.16	(1.05–1.28)	1.47	(1.27–1.69)	30
Men	0.92	(0.80–1.06)	0.95	(0.85–1.06)	1.06	(0.99–1.13)	1.24	(1.13–1.37)	30
*Age-specific mortality*									
0–14 years old	0.83	(0.61–1.14)	0.76	(0.58–0.99)	–	–	1.23	(1.07–1.41)	31
15–64 years old	0.94	(0.80–1.09)	0.97	(0.86–1.10)	1.08	(1.01–1.16)	1.31	(1.18–1.46)	30
≥65 years old	1.14	(0.87–1.50)	1.03	(0.86–1.22)	1.22	(1.10–1.37)	1.53	(1.31–1.80)	30

NCS: natural cubic spline, RR: relative risk, CI: confidence interval, MMT: minimum mortality temperature.

**Figure 2:**
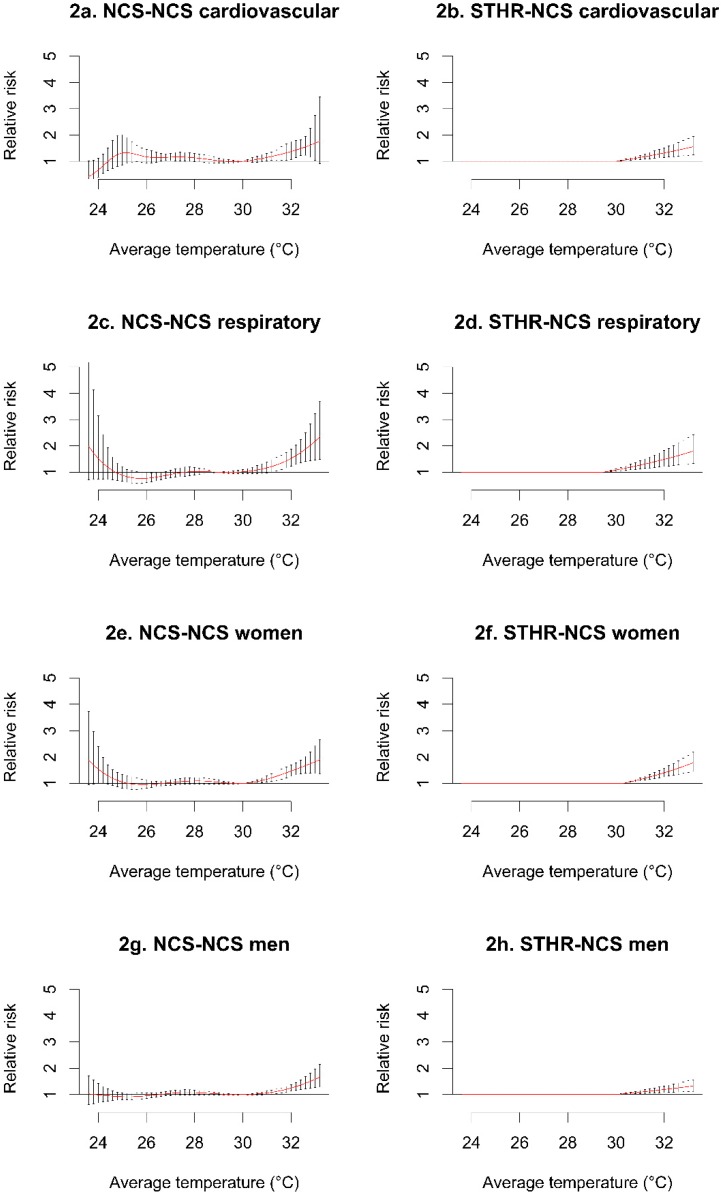
Cause-specific (**a–d**), and sex-specific mortality (**e-h**) relative risk in the NCS-NCS, and STHR-NCS models. The upper thresholds in the STHR-NCS models were based on the respective minimum mortality points. NCS: natural cubic spline, STHR: single high threshold.

**Figure 3:**
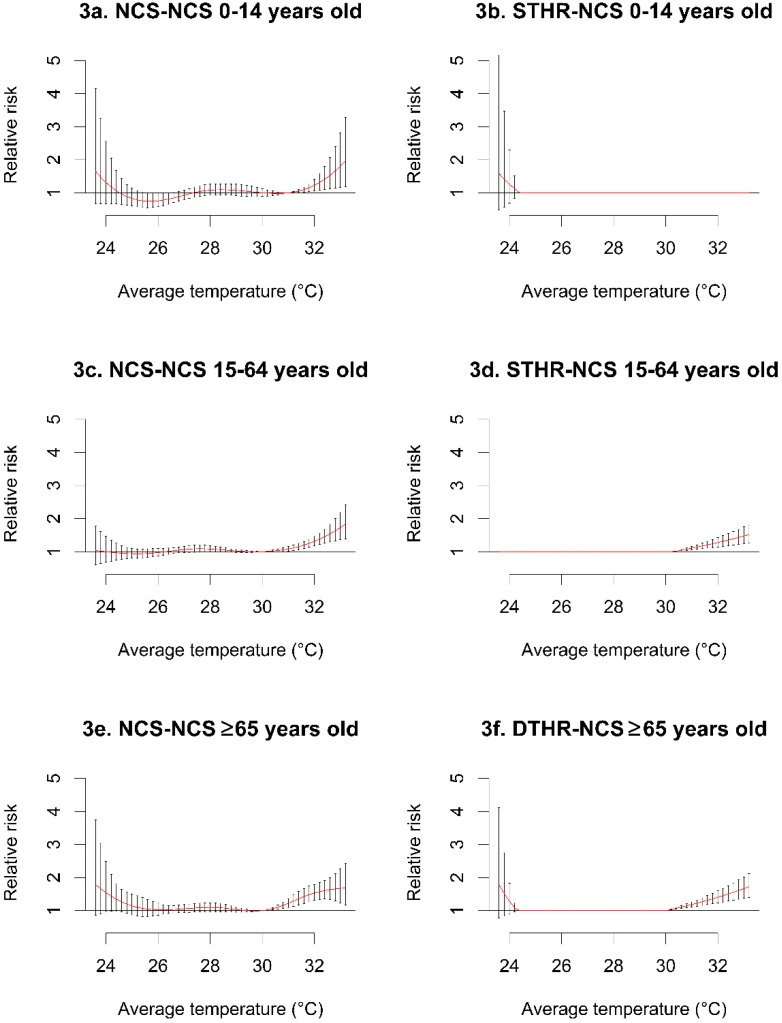
Age-related relative risk in the NCS-NCS, DTHR-NCS, and STHR-NCS models. Only the ≥65 year old category (**e–f**) required a DTHR-NCS model, because of its prominent low and high temperature effects. All other age groups were reduced to STHR-NCS as their final form, with the 0–14-year-old age group (**b**) exhibiting a pronounced lower temperature effect. NCS: natural cubic spline, STHR: single high threshold, DTHR: double high threshold.

Figure 2 and Figure 3 display the simplified models that we derived (right side), which provided a similar or better description of the relationship (vs. the NCS-NCS models on the left side). The only exception was in the mortality in men, where the NCS-NCS model outperformed the STHR-NCS model. In the cause-specific analyses, we observed a higher risk of respiratory-related mortality at higher temperatures, compared to the risk of cardiovascular-related mortality (Figure 2a–d). Women also had a higher risk of mortality at higher temperatures, compared to men (Figure 2e–h).

Figure 3 describes the age-related risk of mortality according to temperature, which was significantly higher in the ≥65-year-old age group. The 15–64-year-old age group (Figure 3d) exhibited higher temperature effects, and the 0–14-year-old age group (Figure 3b) exhibited lower temperature effects.

## 4. Discussion

In the present study, we confirmed that the effects of temperature on mortality in Manila City conform to the non-linear patterns that have been observed in the previous studies in temperate and sub-tropical cities [1,2,5,6,7,8,9,10,15,20,21]. In the present study, the temperature-mortality relationship exhibited a U-shaped pattern with elevated risks at the extremes of the temperature range, with a prominent increase at higher temperatures. In addition, the existence of two points in the model warranted the use of a DTHR-NCS model, which allowed us to assume linearity beyond the threshold points and a null relationship between the points [18]. However, the use of STHR-NCS or DTHR-NCS models instead of an NCS-NCS model created a restricted pattern (unlike the smoothed pattern), although these models provided greater robustness for the specific sub-models in the analyses.

To further quantify the effects of temperature on mortality, we performed subanalyses of the cause-specific, sex-specific, age-specific, and season-specific effects on temperature-related mortality. In most of those models, higher risks were observed at the higher temperature slope, which is consistent with the results of previous studies [1,2,5,6,9,10,20,21]. We also observed a higher risk of respiratory-related mortality in the 99th temperature percentile, compared to the risk of cardiovascular-related mortality. This finding is consistent with those of Ballester *et al*. [22] and D’Ippoliti *et al*. [23], who concluded that hot air during high temperature periods can affect the respiratory function of patients with chronic respiratory diseases. However, we also found that the risk of cardiovascular-related mortality was higher at Lag 3 in the 1st temperature percentile (Figure S1). In this context, Breitner *et al*. [11] have noted that lower temperatures may induce an increase in heart rate and vasoconstriction, which may later cause myocardial infarction due to the decreased myocardial oxygen supply [24]. Furthermore, we found that the risk of respiratory-related mortality was minimal and delayed at Lag 5 in the 1st temperature percentile (Figure S2). Nevertheless, most of the sub-models exhibited a same-day effect in the 99th temperature percentile, except for respiratory-related and 0–14-year-old mortality, which both exhibited delayed effects at Lag 1 (Figures S2 and S3).

Our sex-specific analyses confirmed that women had a higher risk of temperature-related mortality, compared to men; this finding is similar to those of previous studies [23,25,26]. In contrast, studies by Basu *et al*. [27] and Huang *et al*. [28] have reported no significant sex-related differences in the risk of temperature-related mortality. Nevertheless, the sex-related difference that we observed may be attributed to sex-related differences in physiology, and may also be affected by geographical effect modifiers [29,30].

Our age-specific analyses revealed that elderly persons (≥65 years old) had the greatest risk of temperature-related mortality. As the human body ages, its capacity for thermoregulation decreases, which may impair the ability to adapt to heat stress, especially among persons with pre-existing health conditions [23,25,31,32]. Furthermore, we found that the 0–14-year-old age group exhibited a greater susceptibility to lower temperature effects, which is similar to the findings of Gouveia *et al*. [14]. However, the wide confidence interval for this finding may indicate possible effect modifications through underlying disease mechanisms, such as diarrhea [33]. In addition, the age-specific increases in RRs were incremental, with the elderly population having a greater risk across all temperature percentiles.

In the season-specific analyses, we used the log-transformed season-specific mortality to adjust for the very wide confidence intervals that we observed during the initial development. In these analyses, the highest yearly temperatures in Manila City were recorded during the summer season (MAM), which resulted in the higher temperature slope exhibiting the greatest risk of temperature-related mortality (RR: 1.13, 95% CI: 1.05–1.22). During the summer season, many people go outside (e.g., to visit beaches or to travel), which increases their exposure to the sun’s heat. We also observed that SON exhibited a wider low temperature effect at Lag 0. However, this finding is not conclusive regarding the extent of the lower temperature effects, as the confidence interval may indicate potential effect modification due to diarrhea or leptospirosis, which are common during periods with above-average rainfall [34]. Similarly, Chadsuthi *et al*. [35] and Carlton *et al*. [33] have confirmed that rainfall increases susceptibility to diarrhea and leptospirosis. Unfortunately, due to the lack of daily data, we were unable to explore the possible effect modification of these factors on the temperature-mortality relationship.

However, one of the National Epidemiology Center’s [36] reports has confirmed that the incidence of leptospirosis reaches a peak during SON, and that the incidence of acute bloody diarrhea gradually increases from JJA until SON before subsiding in DJF [36]. Diarrhea has a fecal-oral route of transmission through direct/indirect contact with contaminated products/environment [35], and longer incubation periods (several weeks) can lead to death if not treated correctly. Although the recorded cases are usually observed among younger age groups [33], other factors (e.g., the frequency of flooding) can increase the susceptibility of other age groups [35]. In this context, the Philippines experience torrential rains during JJA and SON, due to numerous typhoons. This torrential rain can result in clogged sewers, which results in massive flooding (waist-high in some cases) that may increase the incidence of diarrhea. For example, the NEC report [36] found that 5304 reported cases of acute bloody diarrhea occurred during a single year (52% among 0–14-year-olds, 40% among 15–64-year-olds, 7% among ≥65-year-olds, and 1% among individuals with unknown age). If we assume that the proportions remain fairly constant (with minimal intra-year variation), the majority of diarrhea cases occur among 0–14-year-old and 15–64-year-old individuals, which would likely increase the related mortality rates. We evaluated this assumption by classifying SON mortality using by age groups, and we observed that the greatest effect modification occurred among 0–14-year-old and 15–64-year-old individuals, with the least effect modification among ≥65-year-old individuals (Figure S4). During that same year, 5357 leptospirosis cases were reported, with 9% among 0–14-year-olds, 89% among 15–64-year-olds, and 2% among ≥65-year-olds; the 15–64-year-olds also exhibited the greatest effect modification. However, these patterns have not yet been confirmed using weekly diarrhea and leptospirosis data. Nevertheless, these preliminary assumptions and findings appear to indicate that temperature-related mortality during SON was modified by age via the mechanisms of diarrhea and leptospirosis.

Although the aforementioned season-specific findings that we observed are promising, the national statistics for the Philippines may overestimate or underestimate the effects of diarrhea or leptospirosis in Manila City, due to the national scale of the data and the limited timeframe. Similarly, our assumptions are not robust enough to account for other area-specific variations or the possible interactions of unidentified effect modifiers. Therefore, a longer observation period and a greater number of cases are needed to explore these possibilities.

Possible harvesting during SON was observed in the 5th and 95th temperature percentiles (Figure S5). However, the rainy season (JJA has the highest amounts of rainfall; Figure S6) might have increased the initial risk for persons with chronic illnesses. Thus, it is possible that this increased risk may have carried over to SON (causing the increased mortality), as chronic diseases manifest over time, especially respiratory-related disease. However, we did not have access to data regarding daily rainfall and other parameters that could affect harvesting or explain the wide confidence intervals that were observed during SON and in our entire season-specific analysis.

### Strengths and Limitations

The model development process for this study followed an objectively identified set of steps for model transition based on threshold determination. When we conducted sensitivity analysis for the threshold selection in the all-cause mortality analysis, the new low threshold was not wide enough to capture the low temperature effect, and so we only used the new high threshold for the STHR-NCS. In this context, STHR-NCS is more robust than DTHR-NCS, which explains 80.9% of the variability, and STHR-NCS also facilitates easy interpretation of the findings. These characteristics are desirable for helping to guide policy development, as the field of environmental epidemiology, especially regarding temperature and mortality is a developing discipline in the Philippines. Furthermore, regardless of the changes in the model parameterization for temperature and all-cause mortality, we observed consistently high temperature effects with relatively similar RRs across the models.

This study is limited by the use of specific available meteorological data, and did not consider the daily air pollutant concentrations in Manila City, due to data unavailability. Unfortunately, real-time PM measurements have only recently begun in the Philippines, and the available data does not coincide with the period during which our mortality and meteorological data were gathered. Although some studies have reported a considerable effect for air pollution on the temperature-mortality relationship [37], we believe that air pollution had minimal effects in the study. For example, the adjusted RRs of mortality in São Paulo (a topographically similar city in Brazil) taking into account PM_10_ (RR = 4.63) and ozone (RR = 4.35) are still higher compared to the overall effects estimate observed in the study at the 95th temperature percentile (RR=1.07) [38]. If air pollution were to be considerably influential, then the effect estimates would have been amplified due to the synergistic relationship with temperature, which was not observed in this study [39]. Similarly, ozone effect is mediated by temperature, and given a cross-sectional level of volatile organic compounds or other pollutants, the direct heat effect and the indirect effect through ozone would be captured in this study as a comprehensive heat effect.

This is the first study to use DLNM analysis to evaluate the temperature-mortality relationship in the in Manila City, the Philippines. The study design allowed for a comprehensive analysis of the risk of temperature-related mortality that could be attributed to specific causes, sexes, age groups, and seasons. Therefore, our findings may be useful for developing early warning measures for city-specific responses in Manila City. However, caution should be exercised before extrapolating our findings to other areas in the Philippines, as different cities or municipalities are most likely to have unique temperature ranges, mortality rates, and other area-specific parameters. Further research is needed to verify the harvesting and effect modification by age via diarrhea and leptospirosis in the season-specific analysis.

## 5. Conclusions

We observed elevated risks of temperature-related mortality in Manila City among elderly persons, women, and due to respiratory-related causes. Therefore, these findings may provide a foundation for developing specific policies to address the effects of temperature on health in Manila City. Furthermore, the patterns that we observed in our seasonal analysis may be useful for designing future studies to evaluate the possible effect modification by age via diarrhea and leptospirosis.

## Supplementary Files

Supplementary File 1
