# Supplementary material for: Evaluating the Effects of Temperature on Mortality in Manila City (Philippines) from 2006–2010 Using a Distributed Lag Nonlinear Model
Source: Int J Environ Res Public Health. 2015 Jun 16;12(6):6842–57. doi: 10.3390/ijerph120606842 (PMC4483734; doi:10.3390/ijerph120606842)
Supplement: Supplementary File 1 [file ijerph-12-06842-s001.pdf]

Supplementary Information

Evaluating the Effects of Temperature on Mortality in Manila City, Philippines from 2006–2010 Using Distributed Lag Nonlinear Model

---

Modeling Approach:

During the initial development, we used an NCS-NCS model with the following parameterization:

$$Log[E(Y_t)] = \alpha + \beta T_{t,l} + ns(date, 7 \times 5) + ns(RHave_t, 3) + as.factor(dow) + hod \quad \text{(S1)}$$

This initial model included the expected daily number of mortalities (Y_t, which followed an overdispersed Poisson distribution), vector of regression coefficients for the crossbasis (β), crossbasis in pre-determined temperature and lag dimensions ($T_{t,l}$), seasonal variations (*date*), daily average relative humidity ($RHave_t$), day of the week (*dow*), and holiday (*hod*). Based on previous studies, we chose 3 degrees of freedom (df) for $RHave_t$, and 7 df per year was used for *date* to control for the seasonal and long-term trends [1,2].The *dow* term is a factor of indicator variables and *hod* is a binary variable. We used the city's annual population as an offset to control for changes in the population size over time.

During the df selection for lag and temperature, we created a matrix of possible combinations, which ranged from 4 df to 15 df for both lag and temperature (1 df increments), and used the Akaike Information Criterion (AIC) to identify the optimal df combination. The optimal combination was 7 df for temperature and 4 df for lag, based on the lowest AIC value. In the crossbasis function, both temperature and lag were set at equally-spaced knots, based on the selected df in their dimensions (temperature: percentile, lag: log values). We initially allowed the centering of the model to default to the mean, in order to determine the MMT. After several model runs, we determined that the all-cause mortality MMT was at 30 °C, which was located at exactly the 80th temperature percentile. Furthermore, we used a 15-day maximum lag period to model the effects of temperature on mortality, based on the methods of previous studies [1,3].

In the NCS-NCS model, we found two prominent minimum points at 25.8 °C and 30 °C, based on visual inspection and the minimum mortality calculation. Because the increased susceptibility on both tails resembled a U-shaped pattern (Figure 1a), we also analyzed it using a DTHR model that was set at both thresholds (the DTHR-NCS combination). This model was created by altering Equation (S1) and fitting the thresholds into Equation (S2), as indicated below:

$$Log[E(Y_t)] = \alpha + \beta_{Low} TLow_{t,l} + \beta_{High} THigh_{t,l} + ns(date, 7 \times 5) + ns(RHave_t, 3) + as.factor(dow) + hod \quad \text{(S2)}$$

The initial threshold values were based on the two minimum mortality points, which occurred at 25.8 °C for the low threshold and 30 °C for the high threshold (Figure 1b). β_{Low} and β_{High} served as the vectors of regression coefficients for the lower and higher temperature thresholds, respectively. In this

study, we chose to refer to “cold” and “hot” effects as “low” and “high” temperature effects, due to the subjective definitions of “cold” and “heat” effects across the globe. To determine the optimal values for the DTHR-NCS analysis, we created combinations of multiple thresholds with 0.1 °C increments for the new low threshold (the lowest range value up to 25.8 °C) and the new high threshold (30 °C up to the highest range value). Based on the AIC values for the multiple threshold combinations, the new low threshold was set at 23.6 °C and the new high threshold was at 30.2 °C. However, given that the minimum temperature value in Manila City was at 23.5 °C, the 0.1 °C difference might not have been able to capture the lower temperature effects, and can be considered negligible. Thus, we used a hockey-stick model with an STHR at 30.2 °C as the final model for temperature and all-cause mortality (Figure 1c).

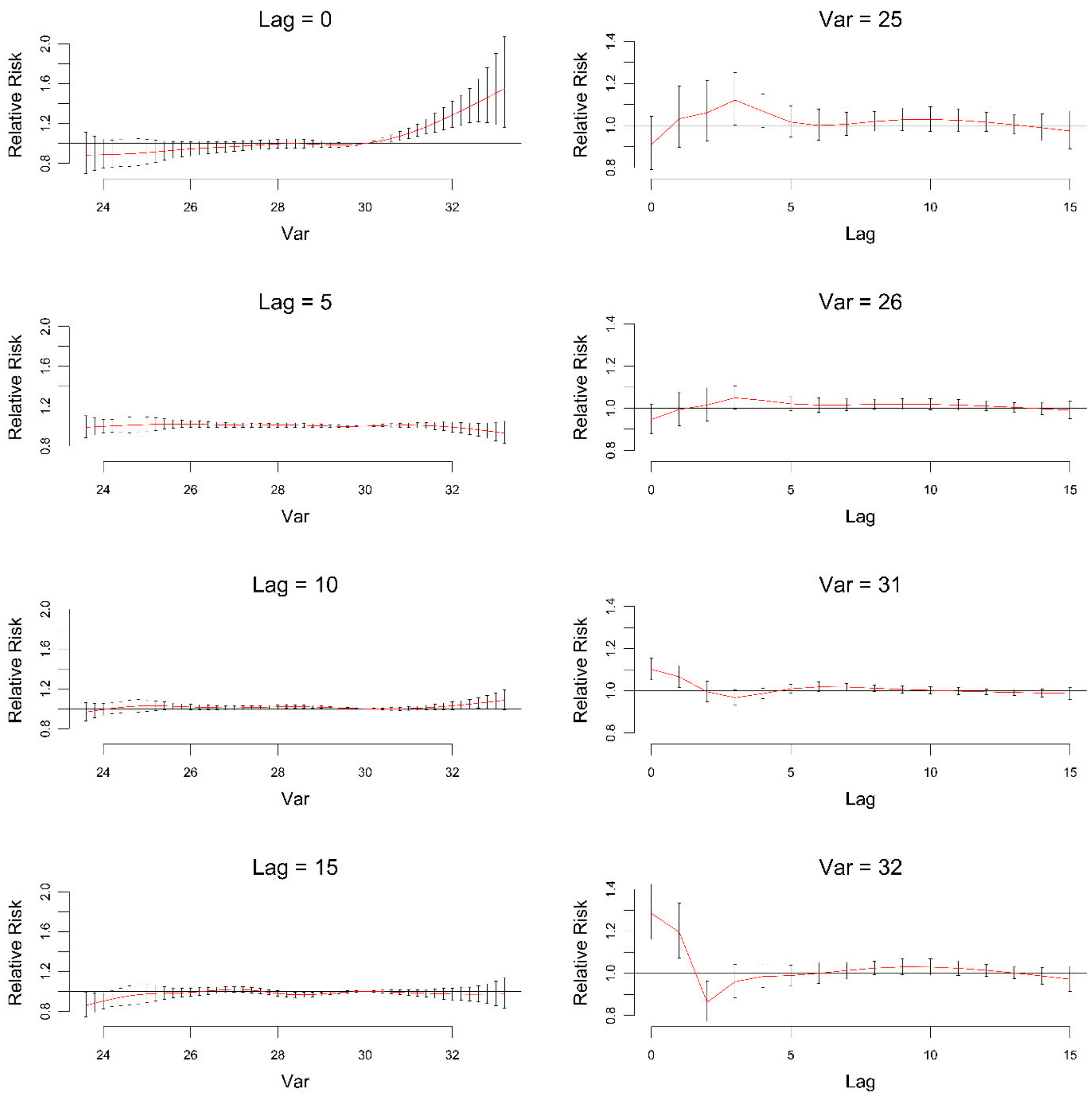

Figure S1. Slices for cardiovascular-related mortality in the lag (**left**) and temperature (**right**) dimensions.

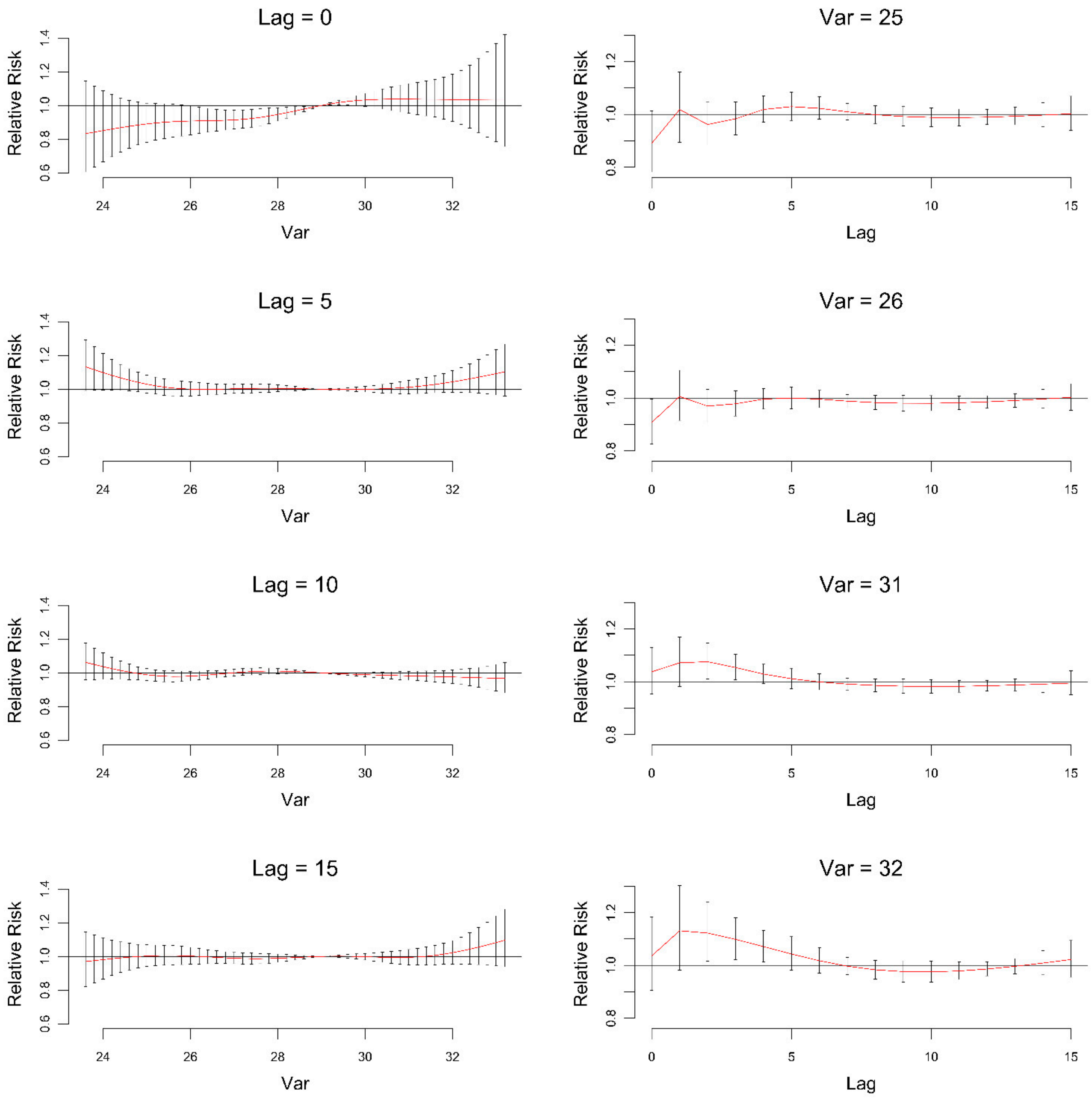

Figure S2. Slices for respiratory-related mortality in the lag (**left**) and temperature (**right**) dimensions.

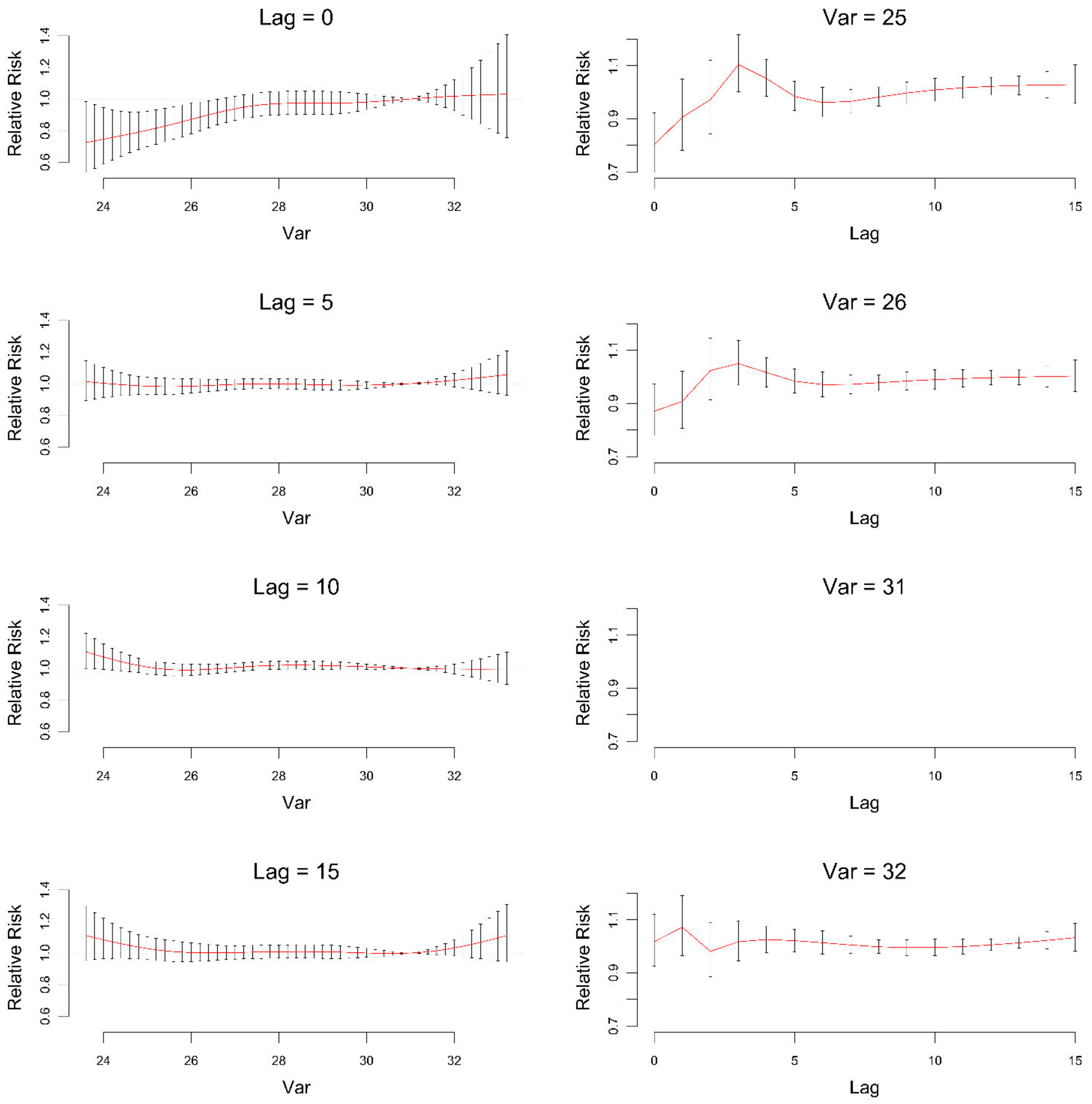

Figure S3. Slices for 0–14-year-old mortality in the lag (**left**) and temperature (**right**) dimensions.

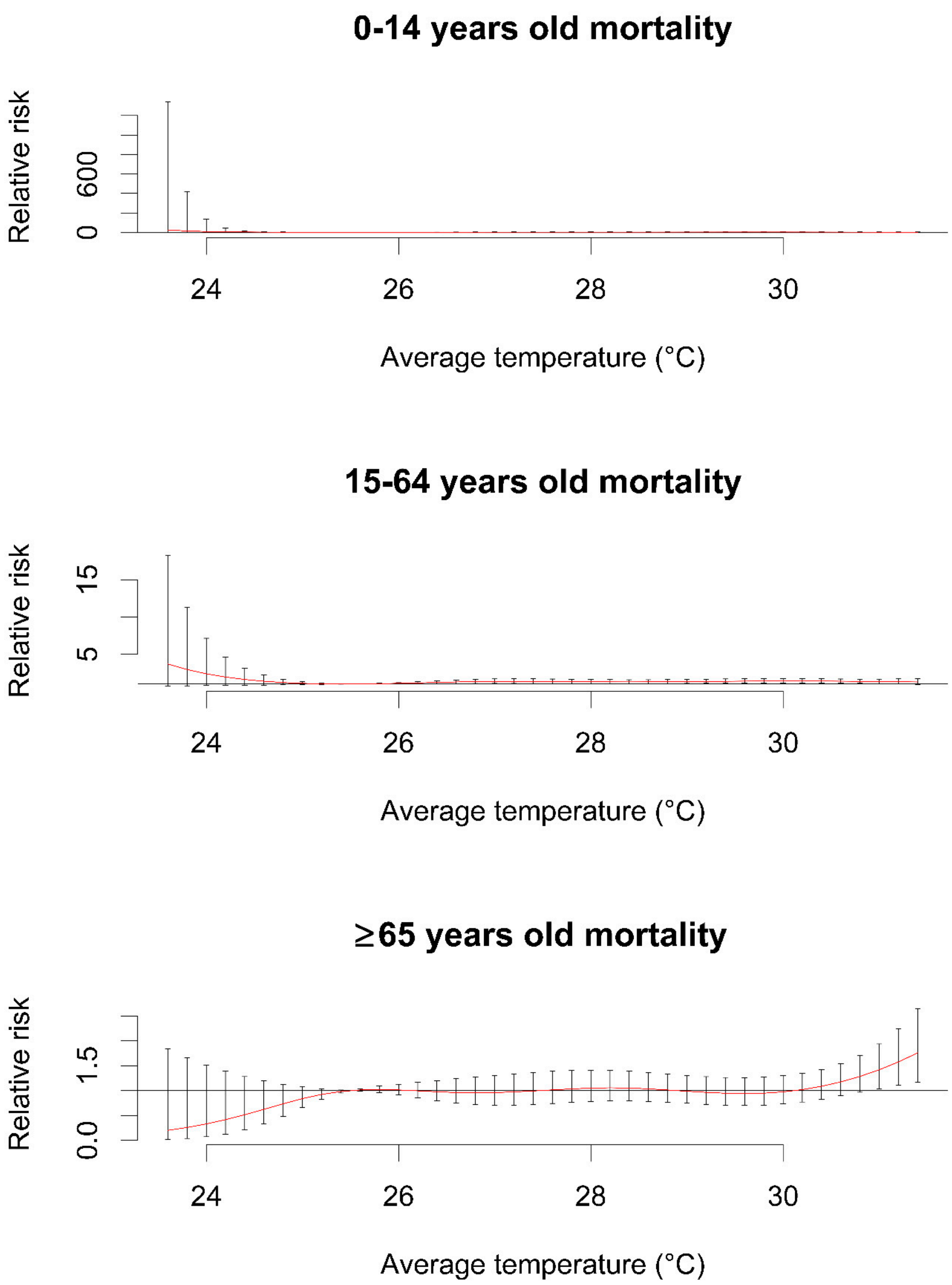

Figure S4. The mortality during September–November according to age; extremely wide confidence intervals were observed for the 0–14-year-old age group, which decreased to the narrowest intervals for the ≥65-year-old age group.

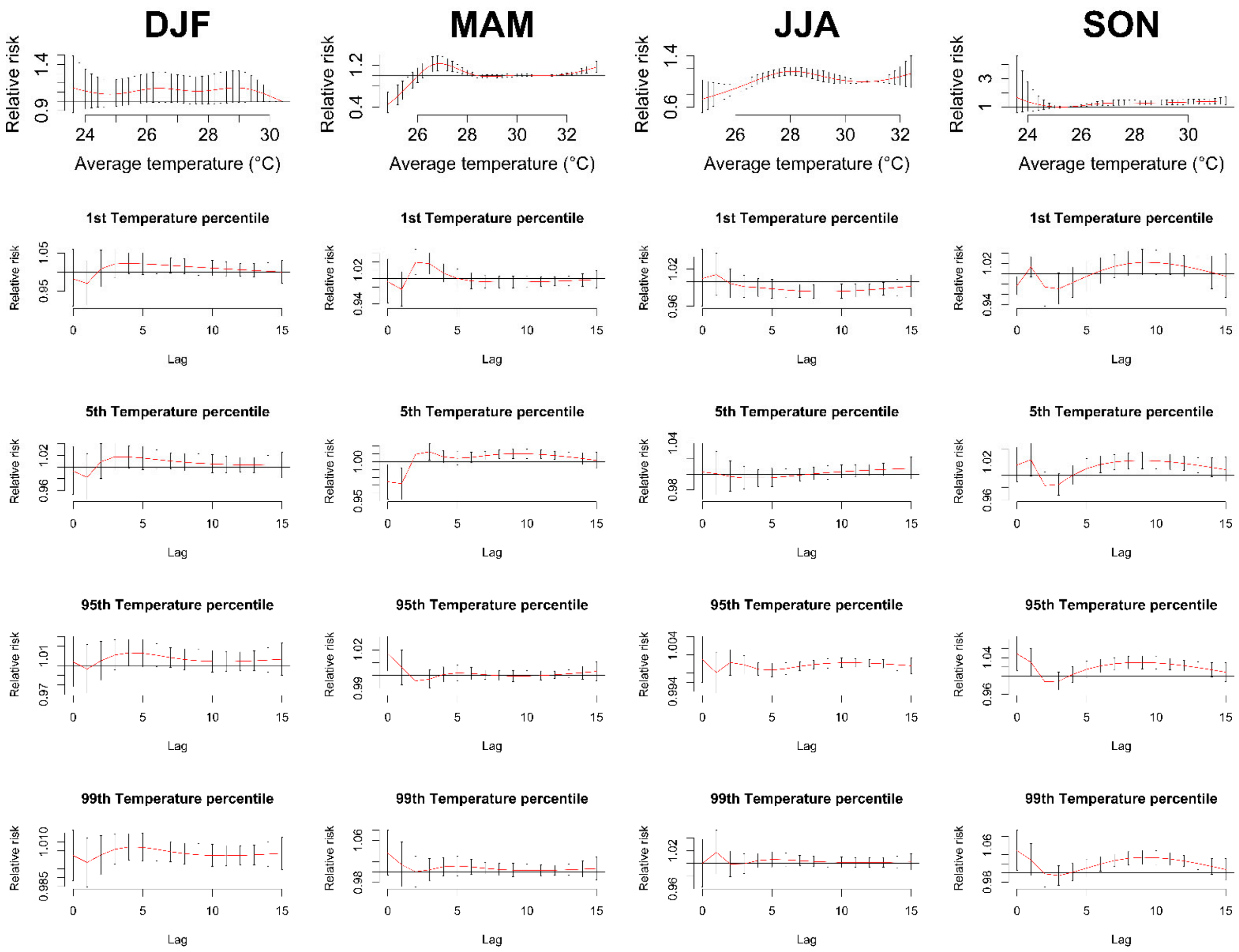

Figure S5. The season-specific relative risks according to the temperature percentiles. The season-specific relationships between all-cause mortality and average temperature are described in the upper row, and the percentile-specific slices are described as indicated. DJF: December to February, MAM: March to May, JJA: June to August, SON: September to November.

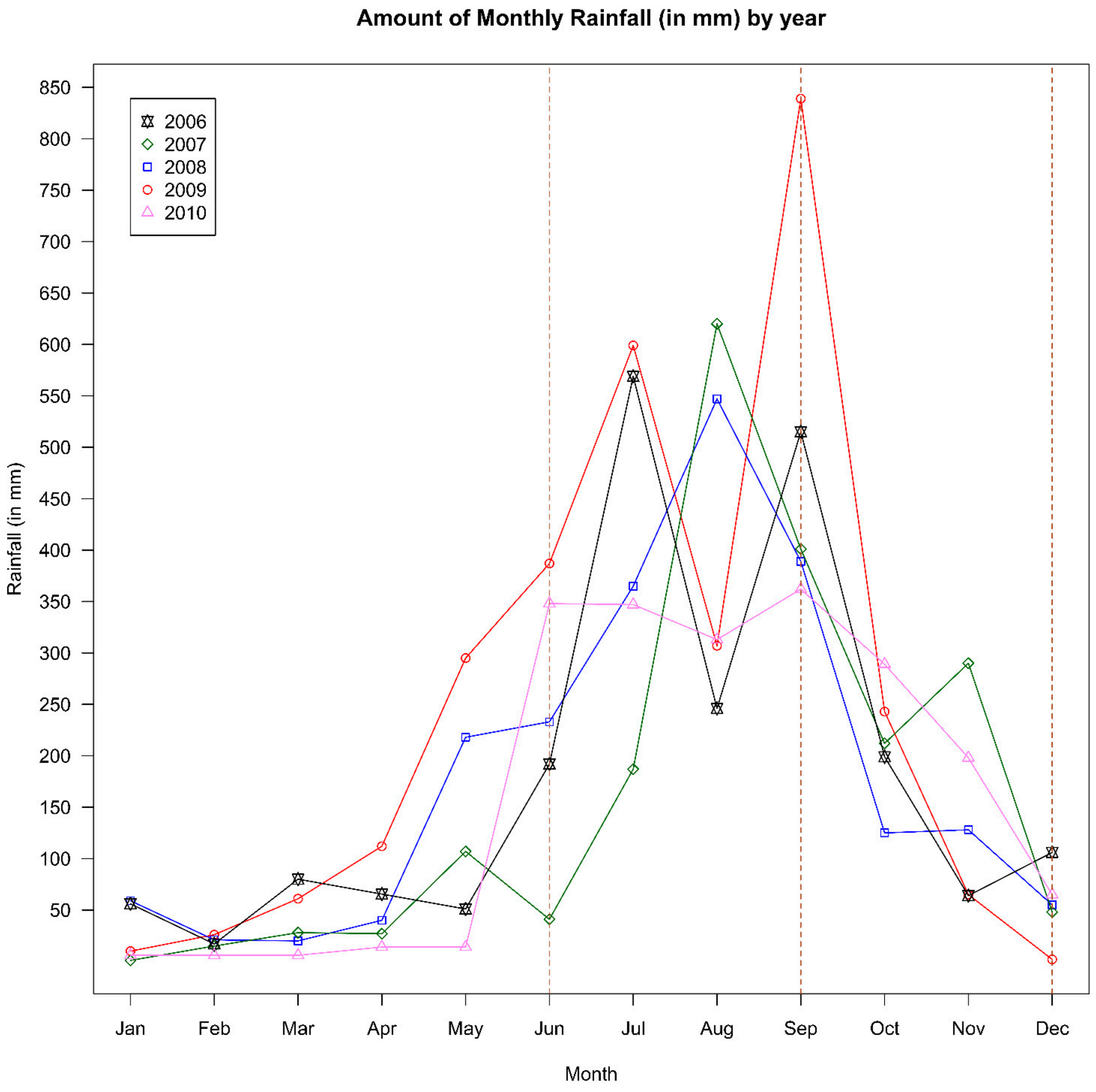

Figure S6. Monthly rainfall in Manila City according to year. The dashed vertical lines indicate the JJA (increasing rainfall) and SON (decreasing rainfall) periods.

References

1. Plavcova, E.; Kysely, J. Relationships between sudden weather changes in summer and mortality in the Czech Republic, 1986–2005. *Int. J. Biometeorol.* **2010**, *54*, 539–551.
2. Ballester, F.; Corella, D.; Pérez-Hoyos, S.; Sáez, M.; Hervás, A. Mortality as a function of temperature. A study in Valencia, Spain, 1991–1993. *Int. J. Epidemiol.* **1997**, *26*, 551–561.

3. Breitner, S.; Wolf, K.; Devlin, R.B.; Diaz-Sanchez, D.; Peters, A.; Schneider, A. Short-term effects of air temperature on mortality and effect modification by air pollution in three cities of Bavaria, Germany: A time-series analysis. *Sci. Total Envir.* **2014**, *485*, 49–61.

© 2015 by the authors; licensee MDPI, Basel, Switzerland. This article is an open access article distributed under the terms and conditions of the Creative Commons Attribution license (http://creativecommons.org/licenses/by/4.0/).
